# Study on buckling of intraocular lens haptic in 2 types of intraocular lens material and its effect on vision

**DOI:** 10.22336/rjo.2020.60

**Published:** 2020

**Authors:** Subhash Joshi Rajesh

**Affiliations:** * Department of Ophthalmology, Vasantrao Naik Government Medical College, Maharashtra, India

**Keywords:** phacoemulsification, intraocular lens material, buckling of the haptic, intraocular lenses biocompatibility

## Abstract

**Aim:** To study the buckling of intraocular lens (IOL) haptics and its effect on vision in patients implanted with either hydrophilic or hydrophobic lens material.

**Study design:** Prospective, observational and descriptive study.

**Setting:** Tertiary eye care center in India.

**Material and methods:** Patients operated for age-related cataract by phacoemulsification technique implanted with hydrophilic or hydrophobic IOL came either for follow-up surgery or complaints in an operated eye, being examined for visual acuity, refraction, anterior segment evaluation, and retinal examination. The position of IOL in terms of optic and haptics was noted and photographed.

**Results:** 317 patients participated in the study. The mean age (SD) of the patients was 65 years (± 4.5). Of 317 patients, 127 (254 eyes) had bilateral, and 190 (190 eyes) had one eye IOL implantation. Out of 444 eyes, 254 (57.2 %) had hydrophilic and 190 eyes (42.8%) had hydrophobic IOL implantation. Buckling of IOL haptic was seen in 37 (8.3%) eyes of which 34 (13.9%) had hydrophilic, and 3 (1.6%) had hydrophobic IOL. Maximum eyes (n=20) with hydrophilic IOL presented during 1-3 years after IOL implantation. Major complaint for which patients came for follow up was blurring of vision (48.9%) and diminished vision (36.3%). Posterior capsular opacification (PCO) was observed in 2/ 190 (1.1%) eyes with hydrophobic and 28/ 254 (11%) eyes with hydrophilic IOL.

**Discussion:** The present study dealt with the long-term performance of the haptics of the hydrophilic and hydrophobic IOLs in relation to its stability in the capsular bag.

**Conclusion:** Buckling of haptic was seen more in the hydrophilic than in the hydrophobic IOLs. PCO was common in these eyes. Change in refraction occurred in eyes with hydrophilic IOL with buckling.

## Introduction

Cataract surgery with implantation of intraocular lens (IOL) has become a standard surgical procedure for age-related cataract. With the significant development in phacoemulsification cataract surgery, surgeons prefer to implant foldable IOLs. The material used for the production of foldable IOL includes hydrophobic and hydrophilic acrylic, silicon and hydrogel. Nowadays, IOLs are increasingly implanted for indications like congenital, developmental, traumatic, complicated cataracts, and refractive lens exchange procedures. The selection of IOL material to be implanted depends on the clinical performance experienced by the surgeon.

The long-term performance of IOL is based on numerous factors like IOL design and material, complications and surgical techniques, biocompatibility of the implanted material in the bag and ocular structures. Opacification of the posterior capsular (PCO), equatorial epithelial cell growth, phimosis of the opening of anterior capsular and recurrent uveitis has been seen if the IOL material is not biocompatible. The biocompatibility of IOL is determined by its resistance to change the optical and mechanical properties due to folding and compression during delivery into the anterior chamber, refractive stability in long-term, injection through small incision and avoidance of PCO [**1**]. Hydrophobic acrylic material has been substantiated to be the most biocompatible material [**2**,**3**]. 

A mechanical property of an IOL is dependent on foldability of its optic and haptics during injection into the capsular bag and unfolding after it is injected. Once an IOL is positioned in the capsular bag, it should withstand forces exerted on it without deformation. Haptics are vital for the proper fixation of IOL in the capsular bag.

In vivo performance of IOL is checked by haptic compression force, compression force decay and axial displacement. Lane et al. studied these characteristics with various IOLs under the laboratory conditions [**4**]. Bozukova et al. also studied biomechanical and optical properties of commercially available hydrophobic foldable IOLs [**5**]. However, there is a paucity of literature on the long-term performance of the haptics of the IOLs after implantation in the capsular bag.

The study presented deals with the buckling of haptics of the IOL in two types of commonly used IOL material and its effect on the visual outcome in the long-term follow up. 

## Material and methods 

This prospective, observational and descriptive study was conducted from January to December 2017 at a tertiary eye care center in central India. The study was conducted in accordance with the tenets of the Declaration of Helsinki.

Patients operated for age-related cataract by phacoemulsification technique implanted with hydrophilic or hydrophobic IOL came for follow up either for other eye cataract surgery, routine follow up or complaints in the operated eye, being examined for visual acuity, thorough anterior segment evaluation, refraction, and retinal examination. Patients with anterior capsular phimosis with buckling of IOL haptics, pupil not dilating with mydriatics, unable to bring previous records, operated for subluxated cataract with placement of endocapsular ring, pseudoexfoliation, retinitis pigmentosa, high myopes, posterior capsular rent and traumatic cataracts, were excluded from the study. 

Visual acuity for distance was examined on Snellen’s chart. Spectacle number was verified on auto lensmeter (Tomey, TL-100, Tomey corporation, Japan). Retinoscopy was performed. Existing refraction was checked on autorefractometer, and final refraction was noted.

The position of IOL in terms of optic and haptics were noted and photographed (Imaging system-990 5X Elite, CSO, Italy) after dilatation of pupil with mydriatics (5% phenylephrine and 0.8% tropicamide). The retinal examination was done by the 90D lens and indirect ophthalmoscope.

**Statistical analysis**

The data was entered in an Excel® sheet (Software version 14.1.0 [110310]/ 2011) (Microsoft Corporation, Redmond, WA, USA), and statistical analysis was performed with SPSS version 13.0 (SPSS Inc, Chicago, IL, USA). Mean and standard deviation were used to describe the continuous variables, while frequencies and percentages were used to describe the categorical and binary variables. 

## Results

Three hundred and thirty-six patients came for follow up from January to December 2017. Out of these, 19 patients were excluded from the analysis as they could not meet the inclusion criteria [Pseudoexfoliation n=2, High myopia n=3, unable to bring records n=2, anterior capsular phimosis with buckling of haptics n=1, retinitis pigmentosa and traumatic cataract 1 each, posterior capsular rent with placement of IOL over anterior capsular rim and subluxated lens with endocapsular ring placement 2 each, pupil not dilating n=5]. Therefore, the analysis was done for 317 patients. Mean age (SD) of the patients was 65 years (± 4.5). There were 187 males (59%) and 130 females (41%). Of 317 patients, 127 (254 eyes) had bilateral, and 190 (190 eyes) had one eye implantation of IOL. All 444 eyes had IOL implantation. Out of 444 eyes, 254 eyes (254/ 444, 57.2%) had hydrophilic, and 190 eyes (190/ 444, 42.8%) had hydrophobic IOL implantation.

**Fig. 1:**
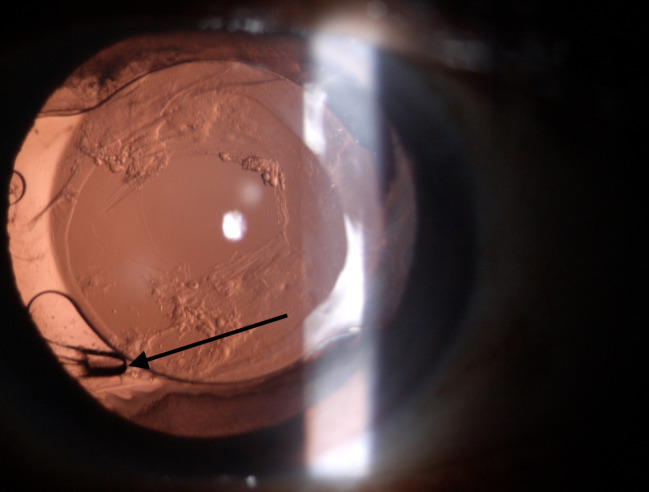
Showing bucking of dual haptic hydrophilic acrylic IOL (Arrow) with central opening of YAG Capsulotomy for opacification of the posterior capsular

**Fig. 2:**
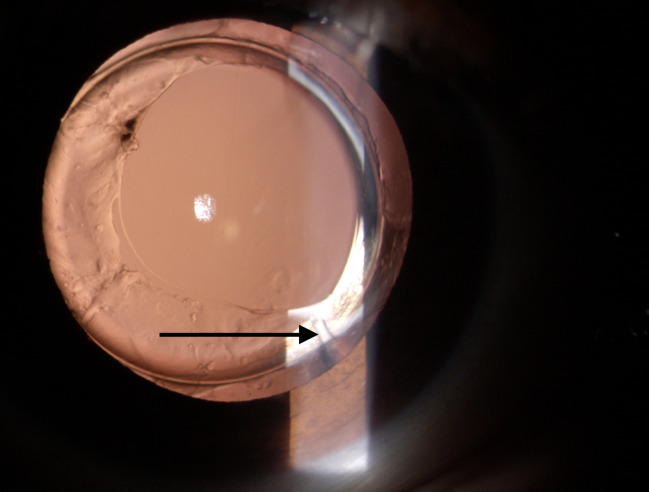
Exhibiting bucking of hydrophobic acrylic IOL (Arrow). Note the clear posterior capsular

**Fig. 3:**
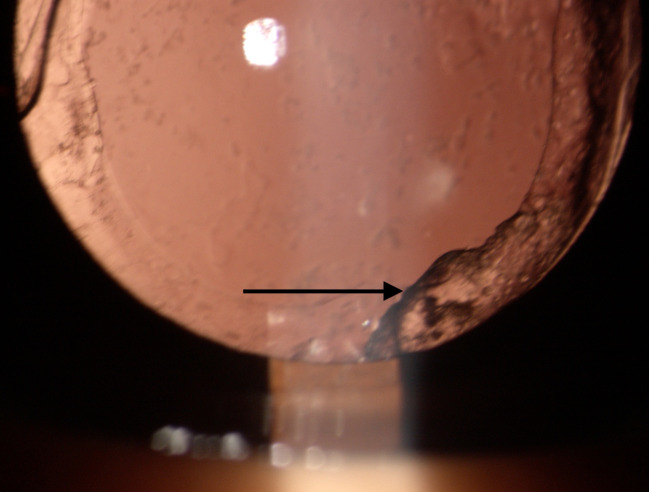
Plate haptic IOL showing buckling of IOL (Arrow) with opacification of the posterior capsular

Buckling of IOL haptic was seen in 37 eyes (37/ 444, 8.3%), of which 34 eyes (34/ 254, 13.9%) had hydrophilic implantation (**Fig. 1**) and three eyes (3/ 190, 1.6%) had hydrophobic IOL implantation (**Fig. 2**). Thirty-three eyes had dual haptic hydrophilic IOL implantation (Acryfold, Appasamy Ocular Devices, Pondicherry, India. Optic diameter of 6 mm and overall length 12.5 mm) and one eye had plate haptic IOL implantation (Ultrasmart, Appasamy Associates, India) (**Fig. 3**). The hydrophobic IOL patients had C-haptic (n=1, CT Lucia 601 PY, Zeiss Optics, Carl Zeiss Meditec AG, Germany, n=2, Supra phob HPNT 200, both lenses with 6 mm optic and 13 mm overall diameter). Buckling was noted in the middle portion of the haptic. Thirty-four eyes had buckling in the vertical direction (6-12 clock direction), while three eyes had it in an oblique direction (2-8 clock direction).

Duration of the IOL implantation and the presentation of the eyes with buckling of haptics are shown in **Table 1**. A maximum number of eyes (n=20) with hydrophilic IOL were presented during 1-3 years after IOL implantation. In patients implanted with hydrophobic IOL, two eyes were presented with buckling of haptic at 3-5 years while one eye presented at 1-3 years after the cataract surgery. 

**Table 1:** Showing duration of phacoemulsification and presentation of patients with buckling of haptics

Duration of the surgery (Years)	Hydrophilic IOL	Hydrophobic IOL
1 -3	20	01
3-5	10	02
> 5	03	-
Total	33	03
@ IOL = Intraocular lens		

Major complaint of the patients during follow up was blurring of vision (155/ 317, 48.9%) and diminished vision (115/ 317, 36.3%). Forty-seven (47/ 317, 14.8%) patients came for follow up and did not have any complaint. No change in the refraction was seen in patients implanted with hydrophobic IOL having buckling of IOL haptics. Whereas patients with hydrophilic IOL implantation, 9 (9/ 254, 3.5%) eyes had buckling of haptics and a change in the refraction from previous spectacle prescription. The observed refractive errors were spherical in 2 eyes (one eye with -1 and other eye with -0.75 spherical), cylindrical in 5 (± 0.5), and both spherical and cylindrical in 2 eyes (Spherical -0.5 and cylindrical -1.0). None of the eyes implanted with hydrophobic IOLs had change in spherical value. One eye with hydrophobic IOL had change in cylindrical (-0.75) value.

**Fig. 4:**
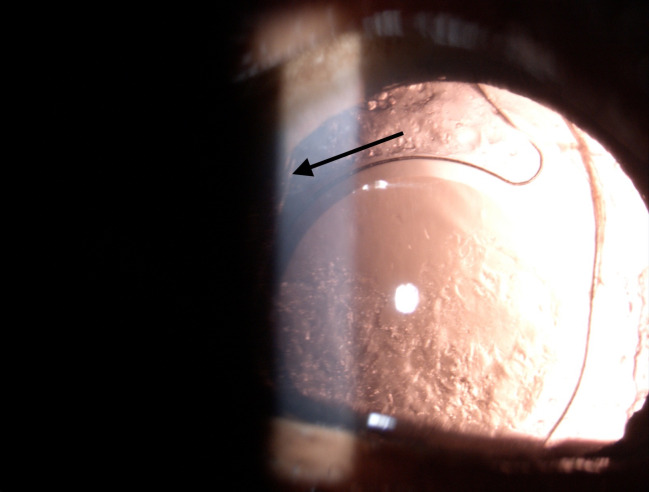
Hydrophobic IOL with buckling of haptic (Arrow) showing posterior capsular opacification

Two eyes with hydrophobic IOL (2/ 190,1.1%) and 28 eyes (28/ 254, 11%) with hydrophilic IOL had PCO (**Fig. 4**). Both the eyes with hydrophobic (Supra phob HPNT 200) and 25 eyes with hydrophilic implantation (one with a plate and 24 with dual haptic) required neodymium-doped yttrium aluminum garnet (ND: YAG) laser capsulotomy. Retinal examination by the 90D lens and indirect ophthalmoscopy was normal in all the eyes. 

## Discussion

For the long-term visual performance, the haptic and optical quality of intraocular lens (IOL) is important. Optic provides clarity in vision while haptic is responsible mechanical and rotational stability after implantation of IOL in the bag. This allows consistency of the A-constant used during IOL power calculation and reduces refractive surprises [**4**]. There have been various experimental studies on the biomechanical and optical properties of the IOL material in regards to its optic and haptic [**4**-**6**]. The hydrophobic acrylic material has been evaluated in various clinical studies and found to be biocompatible [**7**,**8**]. Hydrophilic IOL material is considered to be superior to hydrophobic IOL in terms of uveal biocompatibility [**9**,**10**]. However, it is considered less biocompatible as far as PCO is considered [**10**].

The present study dealt with the long-term performance of the haptics of the hydrophilic and hydrophobic IOLs in relation to its stability in the capsular bag. The haptics of hydrophobic IOLs was found to be stable as only three eyes had buckling (1.6%), against hydrophilic IOLs (13.9%). The strength of the various IOL haptics was measured by Bozukova et al. in an experimental study by haptic compression force assuming the capsular bag size had 10 mm. They found that the C-loop haptic of a single piece lens has high bending deformation capacity to allow IOL to adapt to all capsule sizes [**5**]. This provides perfect rotational stability to the IOL in the long run [**7**,**11**]. Our findings were also in agreement with this, as buckling of the haptic was seen more in hydrophilic (n=33) than hydrophobic (n=3) IOLs. Nowadays, astigmatic correcting (Toric) and multifocal lenses are available in the hydrophilic platform. The rotational stability of these IOLs is important. The shift in effective lens position may compromise long-term performance of toric and multifocal lenses. In our case series, none of the patients had these lenses. 

The exact reason for the buckling of IOL haptic could not be ascertained. The rim of capsulorhexis was found to be intact and free from the fibrosis in most of the eyes except in-patient implanted with the hydrophobic IOL (CT Lucia 601 PY). The fibrosis was prominent near the buckled haptic. However, there was no opacification of posterior capsule. In patients with hydrophilic IOL, rim of the capsulorhexis was not fibrosed. But buckling of the haptic was seen in the middle portion of the haptic. Only one haptic was buckled. We hypothesized that localized fibrosis in the equatorial region of the bag led to the bend in the portion of the haptic; touching that area could be the possible cause of buckling. Another reason could be the softness of the material due to high water content. This makes the hydrophilic material more moldable in comparison with hydrophobic IOL. 

Clinical photography may not be the ideal way to study haptic buckling. Ideally an Ultrasonic bio microscopy should be done to study the change in the effective lens position. However, this facility was not existent in our center. 

In most cases, buckling (n=34) was seen in the vertical direction, i.e., 6-12 clock direction. Only three eyes had it in the oblique direction. Phacoemulsification cataract surgery is performed nowadays through the clear corneal temporal incision; after dialing in the capsular bag, the haptics of the IOL is placed in 6-12 clock direction. This may be the explanation of buckling of haptics in the vertical direction.

Does superior clear corneal incision have a buckling effect in the horizontal direction (9-3 o’clock direction) that needs to be studied either prospectively or retrospectively? 

In the era of polymethyl methacrylate IOLs that are placed in the sulcus or eyes implanted with multipiece IOLs placed in the capsular bag, there has been no mention of buckling of haptics. Thus, capsular bag or stiffness of the haptics has some role to play in the pathogenesis of the buckling of the haptics. Another reason could be biocompatibility of the hydrophilic material, which is lesser biocompatible than the hydrophobic material. Presence of opacification of posterior capsular (PCO), which was seen in 28 eyes (11%) in hydrophilic IOL, as against 2 eyes (1.1%) in hydrophobic IOL, supported this finding. The incidence rate of PCO in patients with hydrophilic IOL has been reported to be more than hydrophobic IOLs [**12**-**15**]. We proposed the proliferation of lens epithelial cells from the equatorial area of the bag on to the posterior capsule along the gap generated by the buckled haptic as a cause of PCO. The long-term multicenter prospective study needs to substantiate this assumption. 

Evaluation of the duration of the cataract surgery and presentation of the patient with buckling of haptics has shown that hydrophilic IOL eyes presented earlier than the eyes with hydrophobic IOL. A total of 20 eyes with hydrophilic IOL implantation were presented 1-3 years after cataract surgery by phacoemulsification technique, while two eyes with hydrophobic IOL were presented 3-5 years after implantation. Early presentation of patients with hydrophilic IOL could be due to the development of opacification of the posterior capsule or change in refraction in these eyes. However, it is difficult to decide the exact duration of the buckling of haptic as this needs to be repeated after cataract surgery, which is impractical. 

Buckling of IOL haptics along with fibrosis of the anterior capsule has been observed in patients operated for cataract associated with retinitis pigmentosa, pseudoexfoliation, uveitis, high myopias and traumatic cataract. Therefore, these patients were not considered for the study purpose [**16**,**17**]. Eyes with non-dilating pupils (n=5), where the haptics of IOL could not be seen, were excluded from the study. Cataract associated with co-morbid conditions and the type of IOL implanted could be found out from the old records. Patients unable to bring old records were excluded from the study.

The blurring of vision (155/ 317, 48.9%) was the major complaint amongst the patients who came for follow up. The diminished vision was a second common complaint (115/ 317, 36.3%). It could be due to the development of opacification of the posterior capsule or change in the spectacle power. However, no complaint was seen in 47 patients (47/ 317, 14.8%). In patients with buckling of haptic, blurring of vision was the prominent symptom. In patients with hydrophilic IOL, 9 eyes had a change in the refraction while 28 eyes had PCO. Change in spherical as well as cylindrical components from the previous refraction was seen. The change in the spherical component could be due to the backward displacement of the IOL optic. This was seen prominently with hydrophilic than hydrophobic IOL; one of the patients with hydrophobic IOL with buckling had a change in the spherical value. This suggested bulky haptic of hydrophilic IOL displaced optic in the backward direction. Malleable haptic of hydrophobic IOL does not displace the optic much. The tilt of IOL must have caused a change in the cylindrical value. We could not measure the exact displacement and tilt of the optic as it was beyond the scope of the study. 

Dilated retinal examination by 90 D lens and indirect ophthalmoscopy was normal in all eyes, suggesting buckling of haptics which did not cause any retinal problem. 

Single surgeon operated patients were included in the study. Those patients who did not have records of the surgical procedure could not be evaluated. There was no way to find out the status of the haptics in eyes having non-dilating pupils. 

A large case series study is required to study if there is any way to prevent the buckling of haptics. Does any modification in the haptics particularly with hydrophilic IOL have a role to play in the prevention?

## Conclusion

Buckling of IOL haptic was more common in hydrophilic than hydrophobic eyes IOL. It leads to change in refraction and PCO, which was common in hydrophilic IOLs. It occurred early in hydrophilic than hydrophobic eyes.

**Conflict of Interest**

The author states no conflict of interest.

**Informed Consent**

Informed consent has been obtained from all individuals included in this study.

**Authorization for the use of human subjects**

The research related to human use complies with all relevant national regulations, institutional policies, is in accordance with the tenets of the Helsinki Declaration, and has been approved by the Ethics Committee of the Department of Ophthalmology, Vasantrao Naik Government Medical College, Maharashtra, India.

**Acknowledgements**

None.

**Sources of Funding**

None.

**Disclosures**

None.
